# Effect of bioaugmentation on gas production and microbial community during anaerobic digestion in a low-temperature fixed-bed reactor

**DOI:** 10.3389/fmicb.2024.1365289

**Published:** 2024-03-14

**Authors:** Yunlong Wang, Xiaoya An, Jian Wang, Xinbo Jiang, Xue Li, Jiamin Yin, Weidong Wang, Jin Piao, Hongyan Zhao, Zongjun Cui

**Affiliations:** ^1^College of Agronomy, Yanbian University, Yanji, China; ^2^College of Integration Science, Yanbian University, Yanji, China; ^3^College of Agronomy and Biotechnology, China Agricultural University, Beijing, China; ^4^College of Life Science, Northeast Forestry University, Harbin, China

**Keywords:** bioaugmentation, anaerobic digestion, carbon carrier, methane production, microbial community

## Abstract

Low temperature is one of the limiting factors for anaerobic digestion in cold regions. To improve the efficiency of anaerobic digestion for methane production in stationary reactors under low-temperature conditions, and to improve the structure of the microbial community for anaerobic digestion at low temperatures. We investigated the effects of different concentrations of exogenous *Methanomicrobium* (10, 20, 30%) and different volumes of carbon fiber carriers (0, 10, 20%) on gas production and microbial communities to improve the performance of low-temperature anaerobic digestion systems. The results show that the addition of 30% exogenous microorganisms and a 10% volume of carbon fiber carrier led to the highest daily (128.15 mL/g VS) and cumulative (576.62 mL/g VS) methane production. This treatment effectively reduced the concentrations of COD and organic acid, in addition to stabilizing the pH of the system. High-throughput sequencing analysis revealed that the dominant bacteria under these conditions were *Acidobacteria* and *Firmicutes* and the dominant archaea were *Candidatus_Udaeobacter* and *Methanobacterium*. While the abundance of microorganisms that metabolize organic acids was reduced, the functional abundance of *hydrogenophilic* methanogenic microorganisms was increased. Therefore, the synergistic effect of *Methanomicrobium* bioaugmentation with carbon fiber carriers can significantly improve the performance and efficiency of low-temperature anaerobic fermentation systems.

## 1 Introduction

Low-temperature anaerobic fermentation utilizes microorganisms to break down organic matter and produce methane at low temperatures (<30°C) (Quispe-Cardenas and Rogers, 2021; Jaimes-Estévez et al., 2023). Traditional anaerobic digestion is typically conducted at moderate-to-high temperatures (30–60°C) (Rusín et al., 2021). In comparison with the high-temperature anaerobic process, low-temperature anaerobic fermentation does not require additional reactor heating, which can significantly reduce energy consumption and operational costs (Rajagopal et al., 2017). In a typical anaerobic digestion process, organic acid metabolic methanogenesis is different significantly from hydrogen metabolic methanogenesis in terms of mechanism and environmental tolerance (Liu et al., 2022). Organic acid metabolic pathways account for 72% of the total methane production, while H_2_ and CO_2_ metabolic pathways contribute only 28%. Organic acid metabolism accounts for a higher percentage, but its reaction process requires more microorganisms to participate, so the microbial activity will be significantly reduced at low temperatures, thus affecting the efficiency of gas production (Hupfauf et al., 2018; Nie et al., 2021). Another factor limiting the efficiency of low-temperature gas production is the excessive accumulation of toxic substances (Lu et al., 2023). This is because the low-temperature tolerance of methanogenic microorganisms is weak compared with that of organic acid-producing microorganisms, which leads to the fact that organic acids cannot be decomposed into methane promptly (Holohan et al., 2022; Liu et al., 2023), and this also causes a negative feedback effect in the digestive system (Kotsyurbenko, 2005). So how improving the low-temperature tolerance of methanogenic microorganisms is one of the effective ways to solve the problem of low-temperature anaerobic digestion.

Many previous studies have used bioaugmentation to improve the efficiency of anaerobic digestion (Jang et al., 2018; Lovato et al., 2021). Bioaugmentation is a method for improving degradation reactions by adding specific microorganisms or microbial communities (Li et al., 2018). Application of bioaugmentation to anaerobic fermentation can effectively enhance the degradation of organic matter and increase methane production (Xu et al., 2022; Yan et al., 2023), Therefore, we expect to improve the limitations of low-temperature anaerobic digestion systems using bioaugmentation. Previous studies have shown that *Methanomicrobium* occurs in relatively high abundance in low-temperature anaerobic digestion reactions and shows a strong correlation with methane production (Lv et al., 2019; Yue et al., 2021). Our earlier experimental results showed that *Methanomicrobium* exhibited significant enrichment on carbon fiber carriers (Zhao et al., 2013), which usually have a developed porous structure and a large surface area that can provide abundant attachment points for microorganisms (Abbas et al., 2021). This not only directly increases the number of microorganisms in the reactor, but also provides a comfortable and stable living environment for the microorganisms, which is conducive to the formation of *Methanomicrobium* (Yang et al., 2017; Zhang et al., 2017). Meanwhile, *Methanomicrobium* exhibits high biological activity on carbon fiber carriers, therefore, we consider combining the *Methanomicrobium* bioaugmentation with the carbon fiber carriers to construct an anaerobic digestive system that has a stronger adaptive ability to low-temperature environments.

To investigate whether biological augmentation with *Methanomicrobium* and the addition of carbon fiber carriers can enhance the performance of low-temperature anaerobic digestion systems, we conducted preliminary work involving the isolation and purification of *Methanomicrobium* for use as an exogenous microbial enhancer. Simultaneously, different quantities of carbon fiber carriers were introduced to improve the performance of low-temperature anaerobic digestion and increase methane production. We evaluated the impact of different microbial concentrations and numbers of carbon fiber carriers on the reaction efficiency to determine the optimal dosages. Furthermore, we examined changes in the microbial community within the reaction system to understand the structure and functional characteristics. Our results provide a theoretical basis for optimization strategies in low-temperature anaerobic digestion.

## 2 Materials and methods

### 2.1 Experimental setup and procedure

The experiment utilized a fixed-bed reactor made of synthetic glass (10 cm in outer diameter, 18 cm in height, and an effective volume of 1 L). The reactor was operated under anaerobic conditions with a one-time feed setup.

To regulate the operating temperature, the bioreactor was placed inside a biochemical constant-temperature chamber (Model MIR 254, Sanyo, Japan) maintained at 20°C.

Gas volume was measured using the drainage method, and regular samples were taken from the reactor outlet to determine the concentrations of volatile fatty acids (VFAs), chemical oxygen demand (COD), pH, and methane.

In the reactor, cylindrical carbon fiber fabric was added as a biofilm carrier (outer diameter of 3 cm and a height of 5 cm, supplied by the Japanese Carbon Company in Tokyo, Japan). The height of the carbon fiber fabric and the liquid level were kept consistent. Each cylindrical carbon fiber carrier occupied 10% of the effective volume of the reactor. Three gradients were set: 0, 10, and 20% carbon fiber carriers, labeled cv0, cv10, and cv20, respectively.

### 2.2 Reactants, inoculants, and bioaugmentation strains

The reaction substrate was an artificially synthesized glucose wastewater composed of water, syrup (homogeneity: 70%, solid content: 45%), and industrial feed (mainly protein, fat, and crude fiber). The COD: N:P ratio was maintained at 300–500 5:1 (Zhang et al., 2012).

The initial inoculum was obtained from laboratory-cultured activated sludge that had been long-term acclimated at 20°C. It was passed through a 1 mm sieve to remove larger solids. The resulting inoculum had a total solid (TS) content of 48.82% and a volatile solid (VS) content of 4.56%, sludge inoculum was controlled at 250 g for all treatment groups.

The biological augmentation strain was laboratory-purified L2, which was isolated and purified from the sludge of wetlands in Yanbian Prefecture, Jilin Province, China (latitude 43°45′22″ N, longitude 128°44′10″ E). This strain is Gram-negative, rod-shaped, strictly anaerobic, and belongs to the *Methanomicrobium*, which can produce methane via both H_2_ and CO_2_ metabolism and organic acid metabolism (Liu and Whitman, 2008).

Three different microbial liquid concentration gradients were established, namely 10, 20, and 30%, corresponding to 30 mL, 60 mL, and 90 mL added microbial liquid, respectively. The total volume of microbial liquid added to the culture medium was controlled at 90 mL. The control group used a sterile culture medium without microbial liquid. Finally, artificial glucose wastewater was used to finalize the volume to 600 mL. Each experiment was repeated three times.

### 2.3 Analytical methods

VFAs were determined using high-performance liquid chromatography (LC-MS2020, Japan), COD was measured using a water quality monitoring instrument (Lovi-bond 99731COD, Germany), pH was determined using a compact pH meter from Horiba, and CH_4_ levels were measured using a biogas analyzer (Model ADG, Landtec, United States).

### 2.4 Microbial analysis

Sludge samples were taken from the reactor at predetermined time points, sample DNA was extracted using the MN NucleoSpin 96 Soil DNA extraction kit. Specific amplification of the bacterial 16S V3 + V4 variable region was carried out using the primers 338F (5′-ACTCCTACGGGAGGCAGCA-3′) and 806R (5′-GGACTACHVGGGTWTCTAAT-3′). For archaea, the 16S V3 + V4 variable region was specifically amplified using the primers Arch349F (5′-GYGCASCAGKCGMGAAW-3′) and Arch806R (5′-GGACTACVSGGGTATCTAAT-3′). PCR products were quantitated by gel electrophoresis (ImageJ software), and samples were mixed at a 1:1 ratio based on their mass. DNA was then purified using the OMEGA DNA nucleic acid purification kit. After purification, a library was constructed for high-throughput sequencing, which was provided by Beijing BioMighty Biotech Co., Ltd.

A bioinformatics analysis was performed using the BioMighty cloud platform. Beta diversity distances were calculated using QIIME (version 1.9.1) and alpha diversity analysis was performed using Mothur v. 1.30. The igraph, Hmisc, pheatmap, and vegan packages were used to organize and initially map the data in the R 4.2.1 environment.

## 3 Results and discussion

### 3.1 Performance of low-temperature fixed-bed digesters with different microbial liquid concentrations and carrier volumes

**Figure 1:**
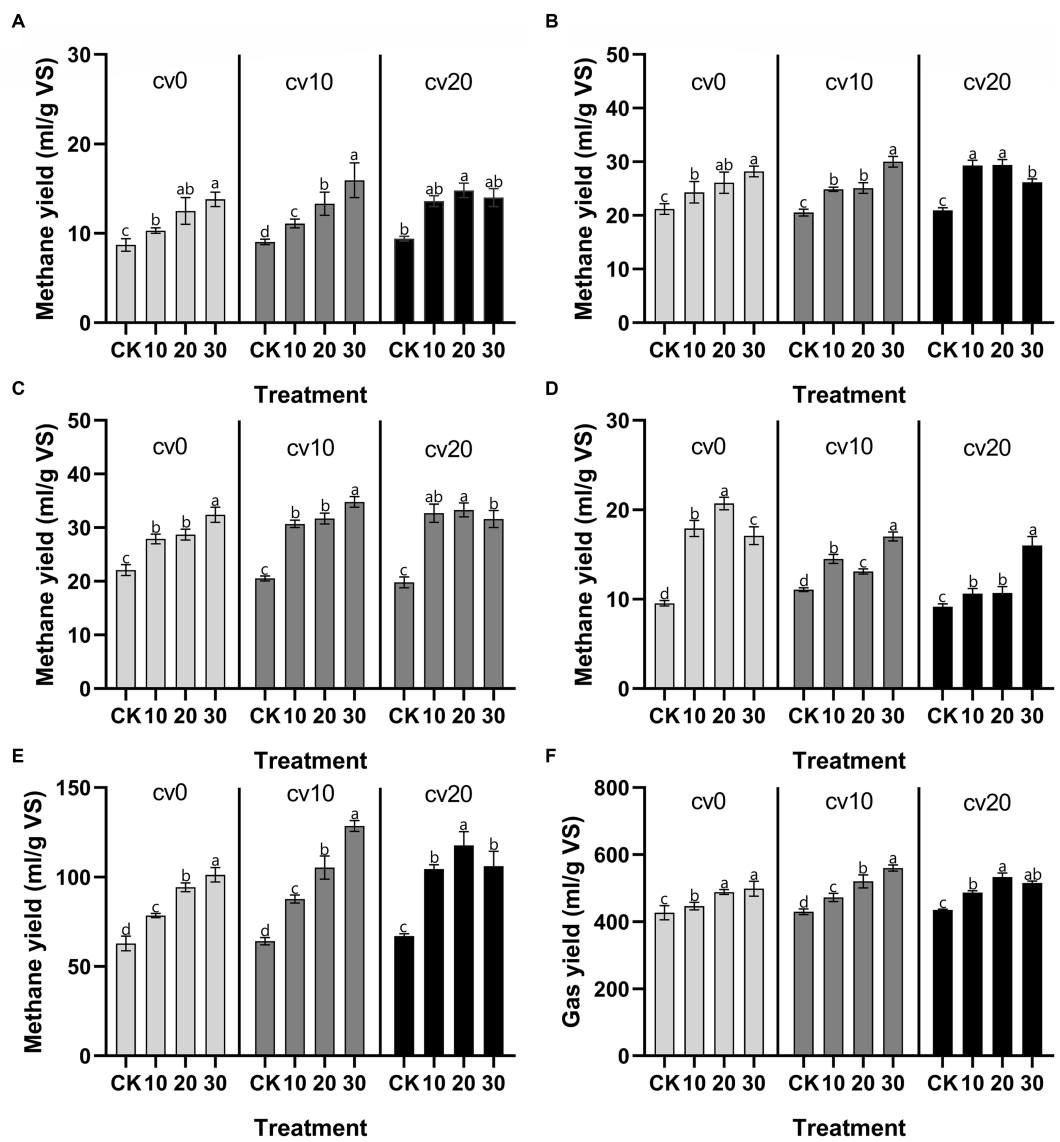
Changes in methane gas production. Daily methane production at 7 days **(A)**, 14 days **(B)**, 21 days **(C)**, and 28 days **(D)**. Cumulative methane production throughout the experiment **(E)**. Cumulative total gas production throughout the experiment **(F)**. cv0 represents the treatment without fiber carriers, cv10 represents a carrier volume of 10%, and cv20 represents a carrier volume of 20%.

Figure 1 compares the performance of low-temperature anaerobic digestion in fixed-bed reactors at different microbial liquid concentrations (control, 10, 20, and 30%) and effective volumes of carbon fiber carriers (0, 10, and 20%). Over a 28 days period, the daily methane production in all treatment groups was higher than that in the control group, and methane production in each treatment group reached its peak on day 21.

As the microbial liquid concentration increased, daily methane production also increased. After the addition of carbon fiber carriers, the daily methane production in the low-concentration microbial liquid treatment group reached a higher level, especially in the first 21 days of the experimental period. In anaerobic digestion processes, low temperatures generally limit bacterial metabolism and reaction kinetics (Zhou et al., 2018); however, the low energy consumption advantage of low-temperature anaerobic digestion makes it valuable for research related to anaerobic digestion in cold regions (Martí-Herrero et al., 2022). Previous studies have shown that the addition of a reasonable amount of exogenous microorganisms through bioaugmentation can effectively optimize the efficiency of anaerobic digestion systems (Martin-Ryals et al., 2015; Li et al., 2022; Linsong et al., 2022). Our results demonstrate that carbon fiber carriers can to some extent reduce the cost of bioaugmentation, implying that carbon fiber carriers can lower the concentration requirements for microbial liquid in fixed reactors while achieving high methane production.

In addition, the cumulative methane production data indicate that bioaugmentation with *Methanomicrobiaceae* effectively enhances the total cumulative methane production in the system and that the cumulative methane production is directly proportional to the microbial liquid concentration. In the treatment group with a 20% carrier volume, the methane production at the highest microbial liquid concentration was relatively reduced; however, in the treatment group with a 10% carrier volume, 30% microbial liquid resulted in the highest methane and total gas production, suggesting that the excessive addition of carbon fiber carriers inhibits the gas production efficiency of the system.

**Figure 2:**
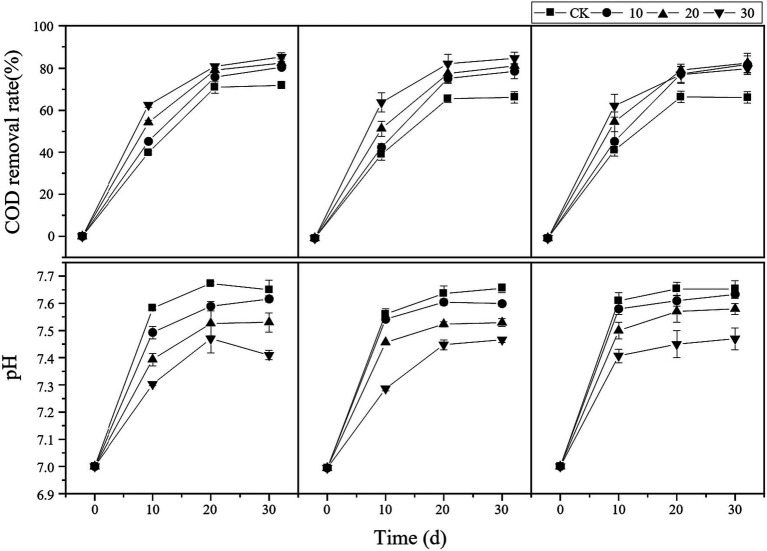
Changes in reactor COD and pH. cv0 represents treatment without fiber carriers, cv10 represents a carrier volume of 10%, cv20 represents a carrier volume of 20%, and different shapes represent different microbial liquid concentrations.

Furthermore, as shown in Figure 2, the COD removal efficiency in all treatment groups was higher than that in the control group at all time points. The COD removal efficiency was directly proportional to the microbial liquid concentration. In the treatment group with a 10% carbon fiber carrier volume and 30% microbial liquid, the maximum COD removal efficiency was 93.48%. In the treatment group with a 20% carbon fiber carrier volume, the COD removal efficiency remained relatively consistent during the latter stages of the experiment. Regarding the anaerobic fermentation process, the pH value in all treatment groups fluctuated between 7.0 and 8.0, which is within the suitable pH range for anaerobic digestion (Zhang et al., 2010). Additionally, the pH value in the treatment groups was lower than that in the control group, indicating that bioaugmentation microorganisms and carrier addition effectively improved the environmental conditions in the reaction system.

**Figure 3:**
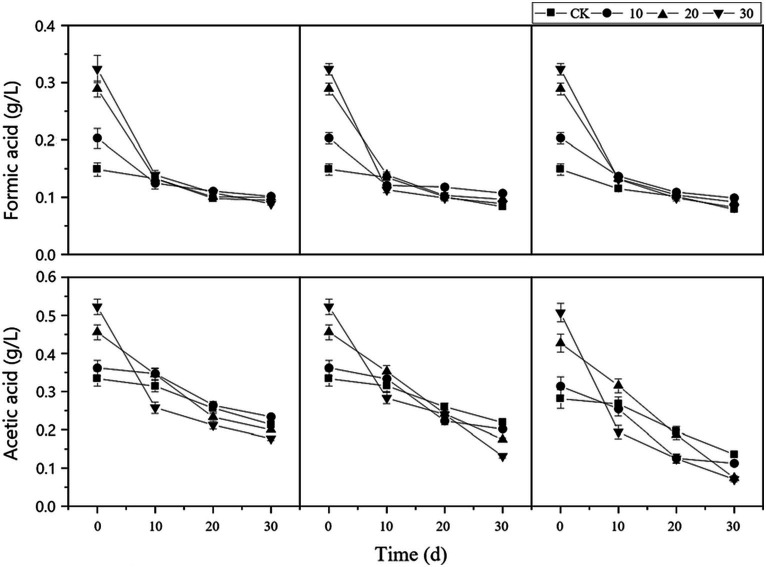
Changes in organic acid concentration in the reactor. cv0 represents treatment without fiber carriers, cv10 represents a carrier volume of 10%, cv20 represents a carrier volume of 20%, and different shapes represent different microbial liquid concentrations.

As shown in Figure 3, the organic acid concentration in each treatment group was significantly higher than that in the control group at the beginning of the reaction. As digestion progressed, the concentrations of formic acid and acetic acid significantly decreased, indicating that the reaction system effectively inhibited the generation of organic acids and maintained them at a relatively stable level.

### 3.2 Bacterial and archaeal community structure in different treatment groups

**Figure 4:**
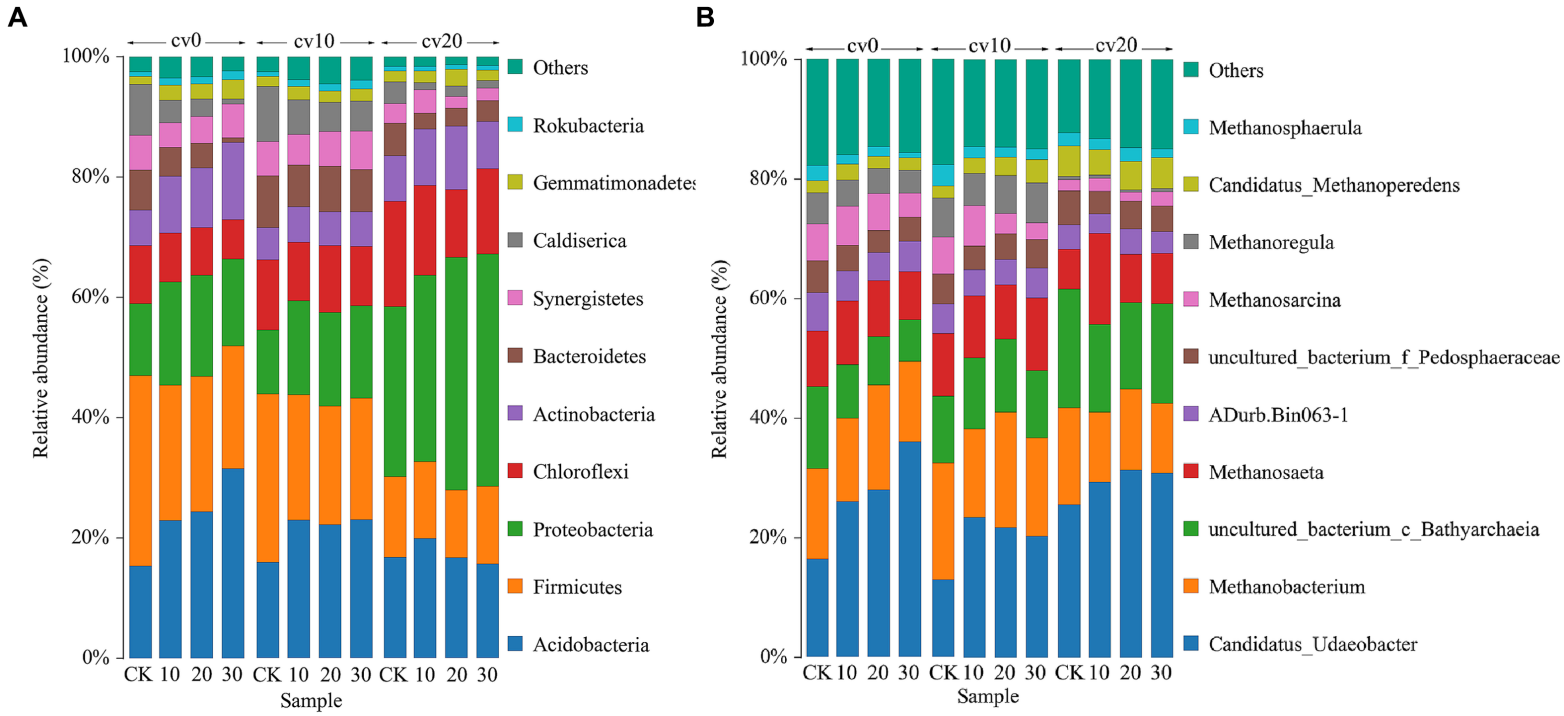
The bacterial **(A)** and archaeal **(B)** community structures in different treatment groups. Different colors represent different species classifications, and species with less than 5% abundance were combined with others.

Figure 4A illustrates the top 10 phyla in terms of abundance in each treatment group. At 0% carrier volume, the abundance of *Acidobacteria*, *Proteobacteria*, and *Actinobacteria* increased with increasing microbial concentration. By contrast, the abundance of *Firmicutes*, *Bacteroidetes*, and *Caldiserica* decreased with increasing microbial concentration. The addition of carbon fiber carriers reduced the abundance of *Acidobacteria* and *Firmicutes* while significantly increasing the abundance of *Proteobacteria*.

Figure 4B displays the archaeal community structure in each treatment group. At cv0, the abundance of *Candidatus_Udaeobacter* increased with the concentration of microbial agents, while the abundance of *Bathyarchaeia* decreased. Previous reports suggest that *Candidatus_Udaeobacter* contains highly affinitive hydrogenases that can metabolize H_2_ to provide energy for the respiratory chain under nutrient-limited conditions (Greening et al., 2015; Willms et al., 2020). On the other hand, *Bathyarchaeia* are known for their autotrophic acetogenic metabolism capabilities (Xie et al., 2022; Hou et al., 2023). This indicates that following the addition of *Methanomicrobiaceae*, which produce methane through H_2_ and CO_2_ metabolism pathways, the microbial community in the reaction system gradually shifts toward these pathways, while the proportion of microorganisms utilizing organic acids to produce methane decreases. These findings are by the organic acid measurements, further demonstrating that bioaugmentation with *Methanomicrobiaceae* effectively reduces the biological production of organic acids and enhances the efficiency of methane production via H_2_ and CO_2_ pathways. At cv20, the abundance of *Bathyarchaeia* was relatively higher in comparison with that at cv10, suggesting that an excessive amount of fiber carriers can alter the community composition and reduce methane production.

### 3.3 Different treatments exhibit significantly different species

**Figure 5:**
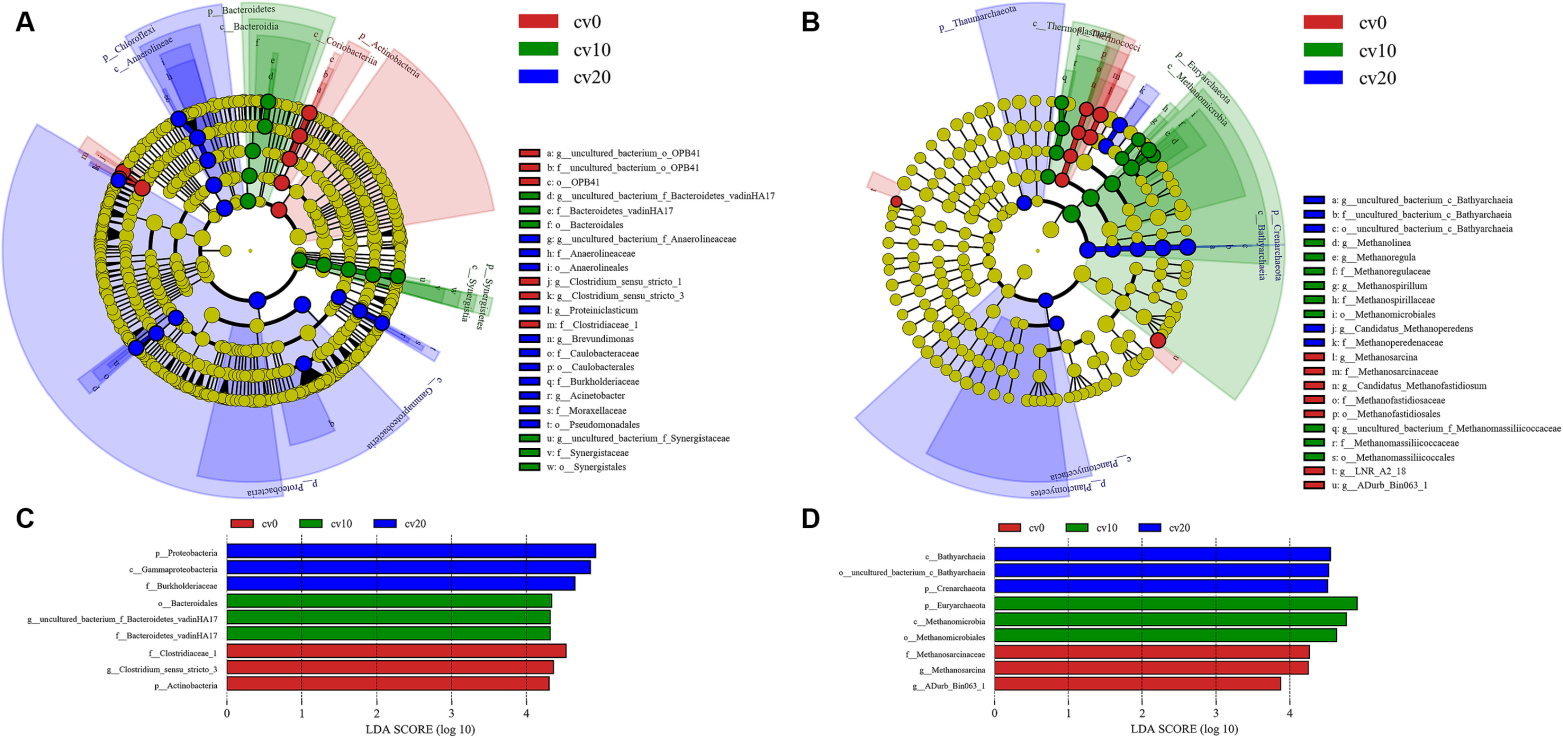
LEFse analysis of bacteria and archaea in different treatment groups. LEFse dendrogram for **(A)** bacteria and **(B)** archaea. LDA scores for **(C)** bacteria and **(D)** archaea.

LEFse was used to assess the phylogenetic tree and LDA scores of bacteria and archaea in different carbon fiber carrier ratio treatments, as shown in Figure 5. The significantly different bacteria in the treatment without carbon fiber carriers included *Acidobacteria* and *Clostridiaceae_1*, which have the highest LDA scores. At 10% effective carrier volume, the significantly different bacteria were *Synergistetes* and *Bacteroidetes*, with *Bacteroidetes* having the highest LDA score. At 20% effective carrier volume, the different bacteria were *Chloroflexota* and *Proteobacteria*, with *Proteobacteria* having the highest LDA score.

The phylogenetic tree of archaea in different treatments is displayed in Figure 5. In all treatments, *Methanobacterium*-related species consistently exhibited a significantly dominant position. This phenomenon was more pronounced at 10% effective carrier volume; while at 20% effective carrier volume, *Bathyarchaeia* was present in more marked abundance. This further validates the earlier results showing differences in acetate and methane production.

### 3.4 FAPROTAX functional gene prediction

**Figure 6:**
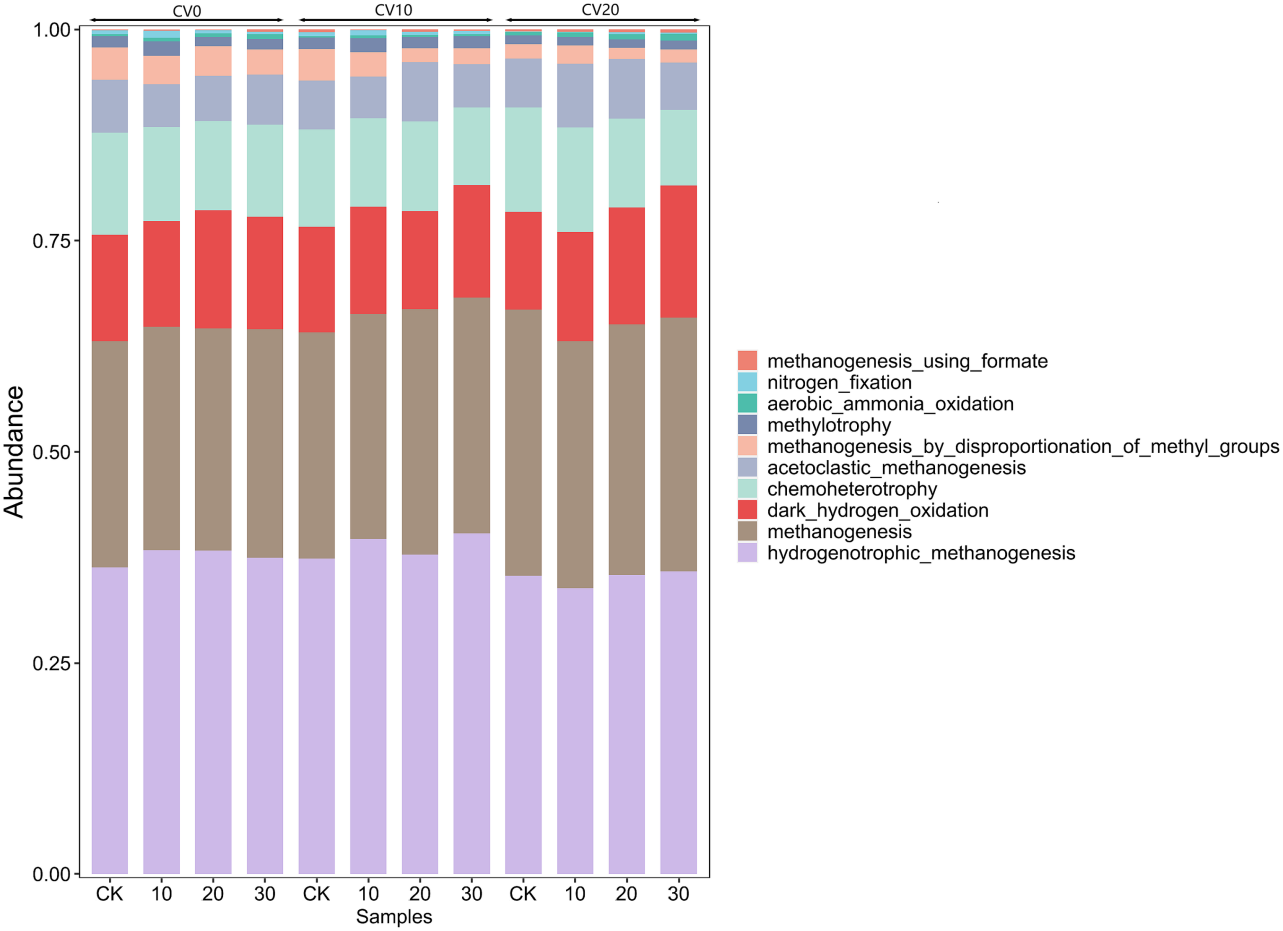
Functional gene prediction for different treatments.

FAPROTAX functional gene prediction can annotate and predict microbial community functions based on the taxonomic classification of 16S sequences. In Figure 6, the horizontal axis represents the samples, and the vertical axis represents the functional groups of the top 10 dominant bacteria in the microbial community. Among these groups, The abundance of hydrogenotrophic_methanogenesis increased to different degrees in all treatment groups, implying that the addition of Methanomicrobium strains significantly improved the methanogenic functioning of the anaerobic digestive system, with 30% exogenous microorganisms and a 10% volume of carbon fiber carrier had the highest functional abundance of hydrogenotrophic_methanogenesis, thus suggesting that the specific biofortification with carbon fiber carrier can maintain stable methanogenic efficiency at low temperatures.

## 4 Conclusion

The addition of 30% methane microbial inoculum and 10% by volume carbon fiber carriers significantly increased the daily and cumulative methane production during anaerobic digestion in fixed-bed reactors at low temperatures. This method effectively reduced COD and organic acids in the system and stabilized the pH. Microbial community analysis and functional prediction indicate that the addition of *Methanomicrobiaceae* inoculum enhanced the abundance of methane-metabolizing microorganisms in the system, reduced the abundance of acetate-metabolizing microorganisms, and increased the proportion of hydrogenotrophic metabolism. These modifications led to higher methane production under low-temperature conditions.

## Data availability statement

The original contributions presented in the study are included in the article/supplementary material, further inquiries can be directed to the corresponding author.

## Author contributions

YW: Conceptualization, Data curation, Formal analysis, Methodology, Software, Validation, Visualization, Writing – original draft, Writing – review & editing. XA: Data curation, Formal analysis, Investigation, Methodology, Supervision, Validation, Writing – review & editing. JW: Data curation, Formal analysis, Investigation, Methodology, Validation, Writing – review & editing. XJ: Data curation, Investigation, Supervision, Validation, Writing – review & editing. XL: Data curation, Formal analysis, Investigation, Methodology, Writing – review & editing. JY: Investigation, Methodology, Supervision, Validation, Writing – review & editing. WW: Project administration, Supervision, Validation, Writing – review & editing. JP: Conceptualization, Methodology, Project administration, Supervision, Writing – review & editing. HZ: Conceptualization, Data curation, Funding acquisition, Methodology, Project administration, Resources, Supervision, Validation, Writing – review & editing. ZC: Project administration, Resources, Supervision, Validation, Writing – review & editing.
